# Blood culture practices in patients with a central line at an academic medical center—Iowa, 2020

**DOI:** 10.1017/ash.2022.45

**Published:** 2022-04-13

**Authors:** Elias Kovoor, Takaaki Kobayashi, Lorinda L. Sheeler, Alexandra Trannel, William Etienne, Oluchi Abosi, Stephanie Holley, Angelique Dains, Kyle E. Jenn, Holly Meacham, Beth Hanna, Alexandre R. Marra, Meredith Parsons, Bradley Ford, Melanie Wellington, Daniel J. Diekema, Jorge L. Salinas

**Affiliations:** 1 University of Iowa Hospitals & Clinics, Iowa City, Iowa, United States; 2 Instituto Israelita de Ensino e Pesquisa Albert Einstein, Hospital Israelita Albert Einstein, São Paulo, Brazil

**Keywords:** Catheter related blood stream infection, Central line associated blood stream infection, Blood culture practices

## Abstract

We analyzed blood-culture practices to characterize the utilization of the Infectious Diseases Society of America (IDSA) recommendations related to catheter-related bloodstream infection (CRBSI) blood cultures. Most patients with a central line had only peripheral blood cultures. Increasing the utilization of CRBSI guidelines may improve clinical care, but may also affect other quality metrics.

The Infectious Diseases Society of America (IDSA) clinical definition for catheter-related bloodstream infection (CRBSI) requires ≥1 set of blood cultures from catheters and ≥1 set from peripheral veins.^1^ However, because cultures obtained from a central line may represent contamination rather than true infection, many institutions discourage blood cultures from central lines. We describe blood-culture practices in patients with a central line.

## Methods

University of Iowa Hospitals & Clinics is an academic medical center with 860 hospital beds. We retrospectively collected data from our health record system for all blood cultures obtained from adult patients (ages ≥18) with any central vascular access (ie, central venous catheter [CVC], peripherally inserted central-line catheter [PICC], port, or Hickman catheter) in emergency department or inpatient units during January–December 2020. The culture source is determined as either central line or peripheral when it is entered into the medical record once collected. We focused on first cultures obtained during each admission because they are usually obtained before antibiotic initiation, and they represent the most important opportunity to diagnose bacteremia. We counted cultures obtained peripherally and through existing central lines within 24 hours of first culture. According to the National Healthcare Safety Network (NHSN) surveillance definition of central-line–associated bloodstream infection (CLABSI), we included central lines inserted ≥3 days before specimen collection.^2^

We classified blood-culture collection as the presence of a CRBSI workup, a non-CRBSI sepsis workup, or an incomplete workup. We defined a CRBSI workup as ≥1 culture from a central line and ≥1 peripheral culture, or ≥2 central-line cultures (according to IDSA guidelines). We defined a non-CRBSI sepsis workup as ≥2 peripheral cultures without cultures from a central line because providers might have suspected secondary bacteremia rather than CRBSI.^3^ We defined an incomplete workup as collections not meeting CRBSI or non-CRBSI sepsis workup. This occurred when only 1 peripheral culture was obtained or when only 1 central-line culture was obtained without peripheral cultures. Although culture of catheter tips is part of IDSA guidelines, this culture is not recommended in our hospital. We have reported the frequency of workup categories stratified by location: (1) emergency department, (2) ward (medicine, surgical, oncology and stepdown units), and (3) intensive care units (ICUs). This study was approved by the University of Iowa Institutional Review Board.

## Results

In this study, we included 1,150 patient admissions with 4,071 blood cultures. Moreover, 349 patient admissions with culture collection (30.8%) met the definition of CRBSI workup (Table 1). Furthermore, 62.8% were deemed non-CRBSI sepsis workups, and 6.9% were deemed incomplete workups. Stratified by location, ICUs had the highest percentage of collections with incomplete workups (8.5%), followed by wards (6.8%) and the emergency department (4.9%) (Table 1).

**Table 1.** Blood Culture Order Practices in a Cohort of 1,150 Unique Patient Admissions at the University of Iowa Hospitals & Clinics, 2020

Blood Culture Practice	Patient Admissions (N=1,150), No.	%		ED (N=452), No. (%)	Ward^a^ (N=414), No. (%)	ICU^b^ (N=284), No. (%)
**CRBSI workup**						
≥1 set peripheral + ≥1 set catheter Or ≥2 set catheter + 0 peripheral	354	30.8	93.6	71 (15.7)	237 (57.3)	46 (16.2)
**Non-CRBSI sepsis workup**						
≥2 peripheral + 0 catheter	722	62.8		359 (79.4)	149 (36.0)	214 (75.4)
**Incomplete workup**						
1 set peripheral + 0 catheter	53	4.6	6.4	22 (4.9)	28 (6.8)	24 (8.5)
1 set catheter + 0 peripheral	21	1.8				

Note. CRBSI, catheter-related bloodstream infection; peripheral, blood culture obtained from peripheral vein; catheter, blood culture obtained from central line; ED, emergency department; ICU, intensive care unit.

a Wards included general medical, surgical, oncology and stepdown units.

b ICUs included surgical and neuroscience intensive care unit (SNICU), medical intensive care unit (MICU), cardiovascular intensive care unit (CVICU), and step-down units.

In total, 204 patient admissions had ≥1 positive blood culture (17.7%). The organisms most frequently isolated were *Staphylococcus epidermidis* (n = 33, 16.2%), *Staphylococcus aureus* (n = 16, 7.8%), and *Escherichia coli* (n = 15, 7.4%) (Table 2). In addition, 33 organisms (16.2%) met the NHSN criteria for CLABSI (Supplementary Table 2). The most frequently isolated CLABSI pathogens were *S. epidermidis* (n = 6, 18.2%), *E. coli* (n = 6, 18.2%), *E. faecalis* (n = 3, 9.1%). *S. aureus* accounted for only 1 CLABSI case (3%).

**Table 2.** Ten Most Common Pathogens From the 204 Patient Admissions With a Positive Culture

Pathogen	Patient-Admissions With at Least 1 Positive Culture, No.	CRBSI Workup (n=47), No. (%)	Non-CRBSI Sepsis Workup (n=106), No. (%)	Workup Nonadherent to Guidelines (n=4), No. (%)
*Staphylococcus epidermidis*	33	13 (27.7)	19 (17.9)	1 (25.0)
*Staphylococcus aureus*	16	5 (10.6)	11 (10.4)	0 (0)
*Escherichia coli*	15	5 (10.6)	10 (9.4)	0 (0)
*Pseudomonas aeruginosa*	8	2 (4.3)	5 (4.7)	1 (25.0)
*Enterococcus faecalis*	8	1 (2.1)	6 (5.7)	1 (25.0)
Vancomycin-resistant *Enterococcus faecium*	6	1 (2.1)	5 (4.7)	0 (0)
*Klebsiella pneumoniae*	6	2 (4.3)	4 (3.8)	0 (0)
*Enterobacter cloacae* complex	4	1 (2.1)	3 (2.8)	0 (0)
*Serratia marcescens*	4	1 (2.1)	3 (2.8)	0 (0)
*Streptococcus anginosus* group	3	1 (2.1)	2 (1.9)	0 (0)

## Discussion

We describe blood-culture practices in patients with central lines. Most patients with a central line had no cultures drawn from their central line. Incomplete initial sepsis workups were infrequent and occurred mostly in ICUs. Obtaining 1 culture set from a central line in addition to a peripheral vein could help better ascertain whether the central line caused the bacteremia, but it may be associated with increased culture contamination and NHSN CLABSI rates.

Studies assessing adherence of blood culture collections with IDSA CRBSI diagnostic recommendations are scarce.^4^ Because central lines are more prone to colonization, some investigators believe that cultures through central lines promote false-positive results and unnecessary antibiotic administration.^5^ Blood cultures including ≥1 from a central line (eg, CRBSI workup) had higher positive rates with skin commensals (*S. epidermidis*) compared to cultures obtained peripherally only (eg, non-CRBSI sepsis workup) (Table 2). Although the ideal number of blood-culture sets for CRBSI is not known, a cross-sectional study among infectious disease providers in 2012 revealed that the most common practice for a CRBSI workup when evaluating febrile ICU patients with central lines was to obtain 2 sets: 1 set of cultures from a peripheral vein and a second set from a central line.^6^ Some experts recommend obtaining 3−4 sets of cultures during a febrile or sepsis episode, especially for critically ill patients (eg, 2 peripheral + 1 catheter) to discern infection from skin contamination. This practice is supported by several clinical trials that have estimated that 2 complete sets of cultures will underdiagnose BSI by ∼15%.^7–9^ Importantly, in our study, the most frequent pathogen detected was *S. epidermidis,* a potential skin contaminant.^10^ In 2016, we created an order set for CRBSI in the electronic record that includes 2 peripheral cultures and 1 catheter culture as a diagnostic stewardship intervention. This order set further assists the diagnosis of CRBSI by reporting differential time to positivity (DTP), as recommended by the IDSA.^1^ Despite the availability of the order set, the frequency at which this set was collected was low (12.1%). In practice, it is difficult to follow IDSA guidelines; methods such as culturing catheter tip and calculating time to positivity are used infrequently.^11^

We used the term ‘regular sepsis workup’ for cases in which at least 2 sets of peripheral cultures were obtained. For example, if a patient had a central line and sepsis, and imaging revealed cholecystitis, this would be the top differential, and providers may order 2 peripheral cultures to identify a pathogen rather than performing a CRBSI workup. Although we were unable to determine whether a CRBSI workup was needed for each patient, CRBSI could be differential for patients presenting with symptoms of an infection while they have an active central line in place. Regardless, ordering only 1 culture either from a peripheral vein or a central line is not advised. With a low number of cultures, contamination cannot be ruled out; hence, we should aim to reduce this practice. Although increasing utilization of IDSA CRBSI blood-culture recommendations may help with clinical diagnosis and management of CRBSIs, it may be associated with an increase in culture contamination, which, in turn, may affect NHSN CLABSI rates. Some investigators have suggested looking at bacteremia, regardless of presence of a central line, as an alternative quality metric.^12,13^ The ideal ratio of CRBSI workups among all cultures obtained in patients with central lines remains unclear.

This study had several limitations. We did not perform a chart review; thus, we were unable to differentiate skin contamination or conduct an analysis stratified by the use of antibiotics. Our analysis did not include cultures ordered directly because we only looked at what was collected. We did not know the intention of the ordering provider; thus, our classification of CRBSI workup may not have fully represented provider knowledge and attitudes toward cultures in patients with a central line. For example, if patients had difficult venous access, nurses may have been unable to execute the complete order and might have drawn fewer cultures than requested. Additionally, because we focused on cultures obtained within 24 hours from the first culture in patients hospitalized with a central line, culturing practices and microbiology results may have varied in subsequent cultures obtained later during hospitalization. Moreover, we could not identify the reason why incomplete workup was highest in ICUs, and further studies are needed to investigate issues leading to collecting 1 culture only.

Analysis of blood-culture data allowed us to characterize utilization of IDSA CRBSI blood-culture recommendations. The impact of increasing utilization of CRBSI guidelines on other quality metrics warrants further investigation.

## Supplementary material

For supplementary material accompanying this paper visit https://doi.org/10.1017/ash.2022.45. click here to view supplementary material
