# Supplementary material for: Blood culture practices in patients with a central line at an academic medical center—Iowa, 2020
Source: Antimicrob Steward Healthc Epidemiol. 2022 Apr 13;2(1):e64. doi: 10.1017/ash.2022.45 (PMC9726581; doi:10.1017/ash.2022.45)
Supplement: Supplementary file 1 [file S2732494X22000456sup001.docx]

**Supplemental table 1:** Pathogens identified in the first 24-hour workup from CLABSI patients reported to NHSN. University of Iowa Hospitals & Clinics, 2020.

| **Pathogen** | **# patients with at least 1 positive culture** | **Percent** |
| --- | --- | --- |
| *S. epidermidis* | 6 | 18.2% |
| *E. coli* | 6 | 18.2% |
| *Enterococcus faecalis* | 3 | 9.1% |
| *Streptococcus anginosus* group | 2 | 6.1% |
| *Streptococcus mitis/oralis* group | 2 | 6.1% |
| Anaerobic gram-negative rods | 1 | 3.0% |
| *Clostridium ramosum* | 1 | 3.0% |
| *Klebsiella aerogenes* | 1 | 3.0% |
| *Lactococcus lactis* | 1 | 3.0% |
| *Pseudomonas putida* group | 1 | 3.0% |
| *S. aureus* | 1 | 3.0% |
| *S. capitis* | 1 | 3.0% |
| *Strep. agalactiae* | 1 | 3.0% |
| Vancomycin resistant *Enterococcus faecium* | 1 | 3.0% |
| Gram positive rods suggestive of diphtheroids | 1 | 3.0% |
| *Klebsiella pneumoniae* | 1 | 3.0% |
| *Proteus miribalis* | 1 | 3.0% |
| *Stenotrophomonas maltophilia* | 1 | 3.0% |

Supplemental table 2: Number of confirmed CLABSIs reported to NHSN based on blood culture workup in the first 24hours of first cultures obtained, University of Iowa Hospitals & Clinics, 2020.

|  | **Frequency confirmed CLABSI reported to NHSN** |
| --- | --- |
| CRBSI workup | 26 |
| Sepsis workup | 17 |
| Non-CRBSI workup | 0 |
